# Adhesion to the extracellular matrix is required for interleukin-1 beta actions leading to reactive phenotype in rat astrocytes

**DOI:** 10.1016/j.mcn.2010.03.013

**Published:** 2010-07

**Authors:** Lauren Summers, Korakoch Kangwantas, Loan Nguyen, Cay Kielty, Emmanuel Pinteaux

**Affiliations:** Faculty of Life Sciences, University of Manchester, Manchester M13 9PT, UK

**Keywords:** Inflammation, Extracellular matrix, CNS, Interleukin, Integrin, Astrocytes

## Abstract

The extracellular matrix (ECM) of the brain is essential for homeostasis and normal functions, but is rapidly remodelled during acute brain injury alongside the development of an inflammatory response driven by the cytokine interleukin (IL)-1. Whether the ECM regulates IL-1 actions in astrocytes is completely unknown. The aim of this study was to test the hypothesis that cellular attachment to the ECM is a critical mediator of IL-1β-induced signalling pathways and development of reactive phenotype in astrocytes. Primary rat astrocytes adhered to fibronectin, laminin and fibrillin-1 in an integrin-dependent manner. Attachment to these ECM molecules significantly increased IL-1β-induced activation of extracellular signal-regulated kinase 1/2 (ERK1/2) and inhibition of RhoA and Rho kinase (ROCK), coincident with loss of focal adhesions and cellular morphological changes. Our data demonstrate that the ECM regulates IL-1 actions in astrocytes via cross-talk mechanisms between ERK1/2 and RhoA/ROCK, which could have important implications in brain inflammatory disorders.

## Introduction

The extracellular matrix (ECM) is essential for the normal regulation of central nervous system (CNS) functions. In healthy brain, the ECM consists mainly of hyaluronan, tenascin-C, and proteoglycans in the parenchyma (Rauch, 2004), whereas more rigid glycoprotein ECM molecules such as fibronectin (FN), laminin (LM), and fibrillins (mainly fibrillin-1, FBN1) are generally restricted to the brain vasculature (Bellail et al., 2004; Jones et al., 2005). However, disruption of the blood–brain barrier (BBB) in response to acute brain injury results in exposure of glial cells to components of the vascular ECM, such as FN, LM and FBN1. Furthermore FN and LM are up-regulated in the brain parenchyma in response to CNS injury (Tardy, 2002; Tate et al., 2007), and plasma FN can extravasate from the vasculature into the brain parenchyma (Sakai et al., 2001). These mechanisms result in resident brain cells being exposed to *de novo* ECM molecules during and after the onset of injury. ECM degradation and remodelling is an important factor in glial scarring (Suzuki and Choi, 1990), which is also characterised by hypertrophy and hyperplasia of astrocytes, a cellular response known as astrogliosis.

ECM remodelling is accompanied by a potent inflammatory response, and a key mediator of acute neuroinflammation is the pro-inflammatory cytokine interleukin (IL)-1. IL-1β (the main secreted isoform) is produced by microglia to act primarily on astrocytes, and we have reported previously that IL-1β induces the production of secondary inflammatory mediators via binding to the IL-1 receptor type 1 (IL-1R1) and downstream activation of the extracellular signal-regulated kinase (ERK1/2) in these cells (Parker et al., 2002). IL-1 is critical for the induction of reactive astrogliosis characterised by morphological changes such as cellular flattening, elongation and stellation, and these effects have been observed both *in vivo* (Giulian et al., 1986; Herx and Yong, 2001) and *in vitro* (John et al., 2004). Recent research demonstrated that deactivation of the RhoA/ROCK signalling pathway is a key signalling event in the development of reactive astrogliosis induced by IL-1 *in vitro* (John et al., 2004). However, the involvement of the ERK1/2 signalling pathway and possible cross-talk mechanisms between the ERK1/2 and RhoA/ROCK pathways in these responses is unclear. Importantly, whether the ECM regulates these responses in astrocytes is completely unknown. Given that the ECM is dynamically remodelled during acute brain injury, the aim of the present study was to test the hypothesis that the ECM is a critical regulator of IL-1β-induced signalling pathways and subsequent development of reactive phenotype in rat astrocytes. Here we demonstrate a novel mechanism whereby attachment to ECM is critical for IL-1β-induced inhibition of RhoA/ROCK and reactive phenotype in an ERK1/2-dependent manner. Our data provide new insight into cross-talk mechanisms between the ECM, ERK1/2 and RhoA/ROCK signalling pathways in the development of reactive phenotype in astrocytes.

## Results

### Astrocytes adhere to the ECM in an integrin-dependent manner

**Fig. 1:**
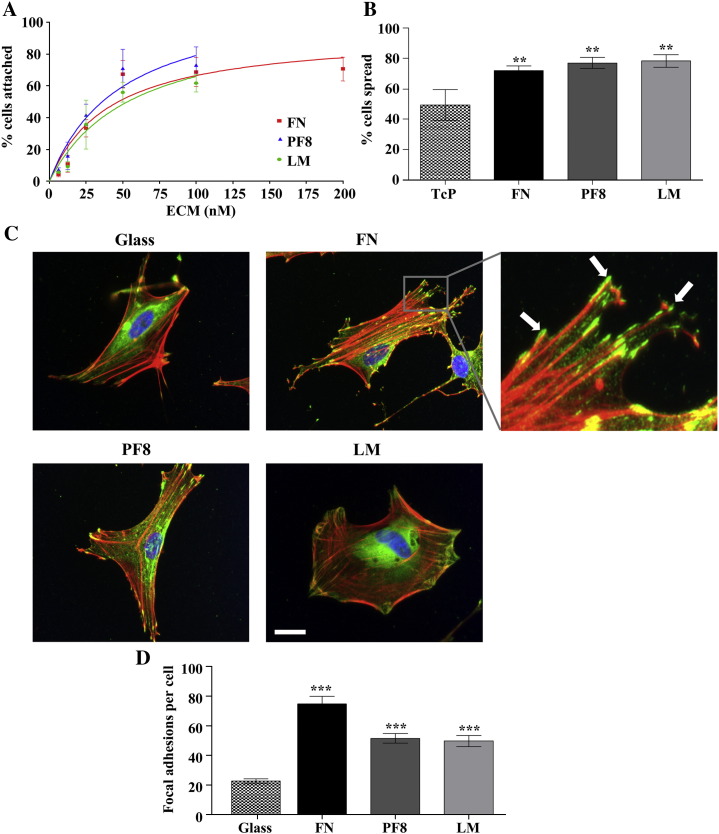
Astrocytes attached, spread and formed focal adhesions on ECM molecules. A. Astrocytes were seeded into wells pre-coated with varying concentrations of fibronectin (FN), FBN1 fragment (PF8) or laminin 111 (LM) for 30 min, and maximal astrocyte attachment occurred at approximately 100 nM with a curve fitted by non-linear fit. B. Cell spreading was assessed by quantification of phase contrast images, where spread cells were elongated and phase dark. Astrocytes were significantly more spread after 150 min in wells pre-coated with 100 nM FN, PF8 and LM compared to TcP. C. Cells were seeded onto ECM-coated coverslips and left overnight before staining with an antibody against vinculin (green), phalloidin to detect actin stress fibers (red) and DAPI nuclear staining (blue). Astrocytes had a more extended morphology and formed significantly more focal adhesions (indicated by white arrows) on coverslips coated with FN, PF8 and LM compared to bare glass. Scale bars represent 20 μm. Data were analysed using one-way ANOVA with Tukey *post-hoc* tests to compare all data sets where *P* < 0.01 ** and *P* < 0.001 ***. Data are the mean ± SD and images are typical of three independent experiments.

**Fig. 2:**
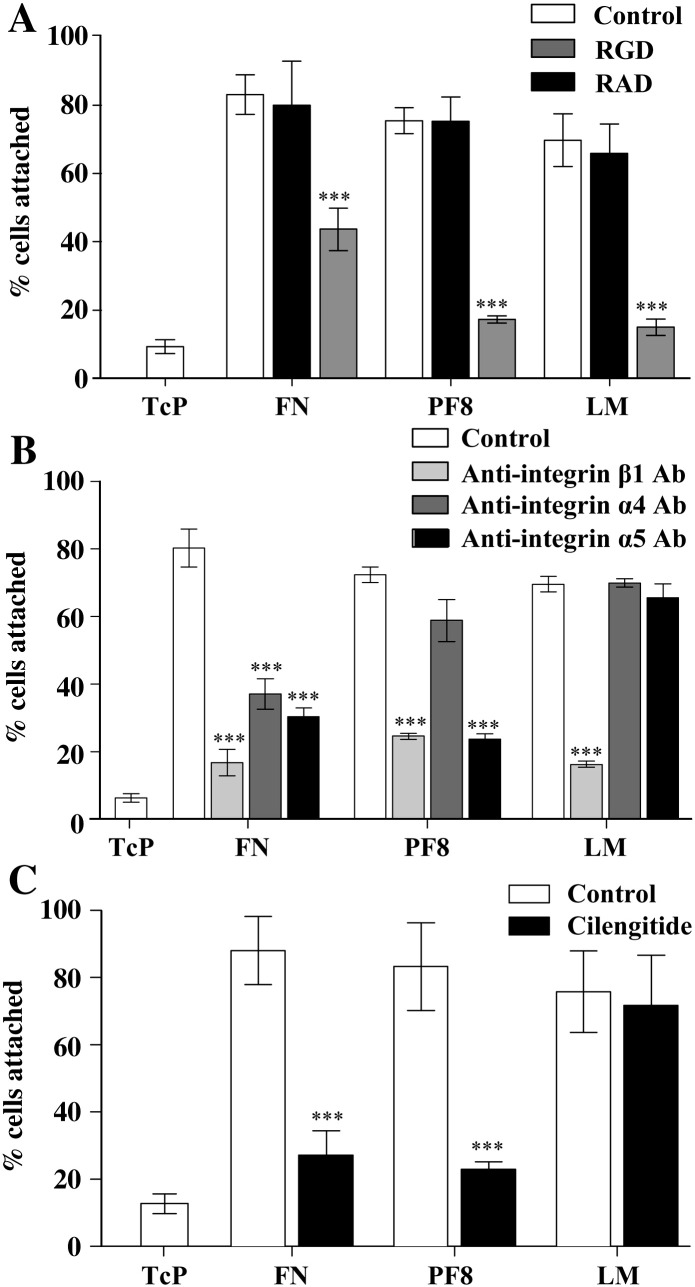
Astrocyte attachment to ECM was RGD and integrin-dependent. A. Astrocytes were pre-treated with RGD peptide or RAD control peptide (10 μg/mL, 30 min) and then seeded into ECM-coated wells for 30 min. The RGD peptide significantly inhibited adhesion to FN, PF8 and LM compared to the RAD control peptide. B. Similarly, astrocyte adhesion to FN, PF8 and LM was significantly inhibited by pre-treatment with anti-integrin antibodies (10 μg/mL, 30 min). C. Pre-treatment of astrocytes with the αv integrin inhibitor, cilengitide (10 μM, 30 min), significantly inhibited adhesion to FN and PF8. Data were analysed by two-way ANOVA with Bonferroni *post-hoc* tests to compare treated cells with untreated control cells, where *P* < 0.001 ***. Data presented are the mean ± SD of three independent experiments.

Astrocytes attached to FN, PF8 (recombinant cell binding fragment of FBN1) and LM in a concentration-dependent manner with maximal binding at approximately 100 nM (Fig. 1A). Astrocytes were significantly more spread (phase dark and elongated) when attached to ECM compared to bare tissue culture plastic (TcP) (Fig. 1B). Astrocytes also formed significantly more focal adhesions when seeded onto ECM-coated coverslips compared to bare glass, shown by localisation of vinculin to the end of actin stress fibers (as indicated by white arrows in Fig. 1C and quantified in Fig. 1D). Treatment with an RGD peptide significantly inhibited adhesion to FN, PF8 and LM, compared to a RAD control peptide, implying that astrocyte adhesion to ECM molecules was integrin-dependent (Fig. 2A). To investigate the nature of the integrins supporting cell adhesion, astrocytes were treated with specific integrin-blocking antibodies at the time of plating onto ECM molecules. Addition of a blocking anti-integrin β1 antibody significantly inhibited adhesion to FN, PF8 and LM (Fig. 2B), whilst blocking anti-integrin α5 antibody significantly inhibited adhesion to FN and PF8, and blocking anti-integrin α4 antibody significantly inhibited adhesion to FN. Cell adhesion to ECM in the presence of integrin-blocking antibodies was, however, higher compared to cell adhesion onto TcP, suggesting that multiple integrins or other adhesion molecules are involved in astrocyte adhesion to ECM molecules. Treatment of cells with cilengitide (a specific cyclic RGD peptide inhibitor of integrin αv) (10 μM) almost completely inhibited adhesion to FN and PF8, but not LM (Fig. 2C).

### Adhesion to ECM results in enhanced and sustained IL-1β-induced ERK1/2 activation

**Fig. 3:**
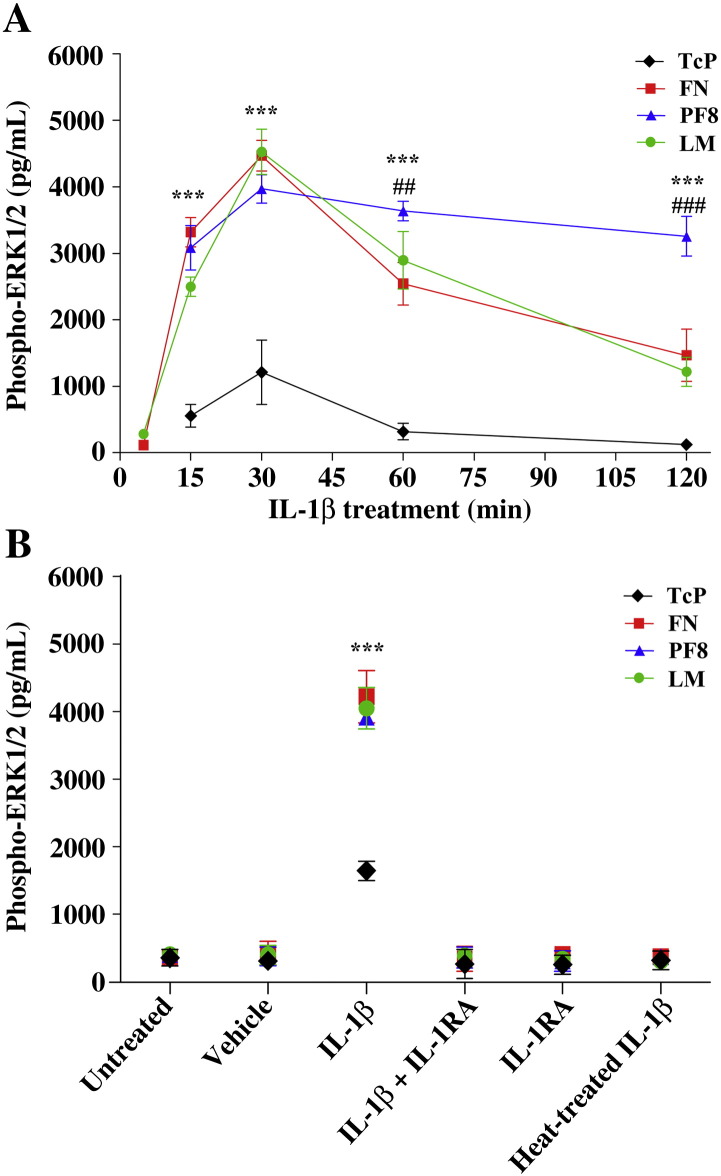
Attachment to ECM enhanced IL-1β-induced ERK1/2 activation. A. Astrocytes were treated with IL-1β (10 ng/mL) over a time course of 120 min, and ERK1/2 activation was assessed by ELISA. Astrocytes showed significantly increased IL-1β-induced ERK1/2 activation when attached to FN, PF8 and LM compared to TcP where *P* < 0.001 ***. ERK1/2 activation was significantly sustained on PF8 following 60 min IL-1β treatment compared to FN, where *P* < 0.01 ##, and on PF8 following IL-1β treatment for 120 min compared to FN and LM, where *P* < 0.001 ###. B. Control experiments showed that untreated cells had very low levels of ERK1/2 activation, the vehicle used to dilute IL-1β did not induce ERK1/2 activation and that IL-1β-induced ERK1/2 activation was inhibited by IL-1RA (10 μg/mL). Again, cells on FN, PF8 and LM had significantly higher IL-1β-induced ERK1/2 activation than cells on TcP where *P* < 0.001 ***. Data were analysed by two-way ANOVA with Bonferroni *post-hoc* tests to compare all data sets. Data presented are the mean ± SD of three independent experiments.

ERK1/2 is a major downstream element of the IL-1β signalling pathway in astrocytes (Saklatvala et al., 1996). Therefore we investigated whether attachment to the ECM modulates IL-1β-induced ERK1/2 activation in astrocytes. In cells seeded on TcP, ERK1/2 activity was significantly increased (by approximately two-fold) 15 min after IL-1β treatment (Fig. 3A). IL-1β-induced ERK1/2 activation was significantly increased at 15 min (by approximately three-fold) when astrocytes were seeded onto ECM molecules compared to cells seeded on TcP. ERK1/2 activation was significantly sustained for up to 120 min in astrocytes seeded onto PF8 compared to TcP, and was also strongly sustained in cells seeded onto FN and LM, although this was not significant. Increased levels of ERK1/2 activation detected in cells adhered to ECM molecules were not a result of increased cell proliferation, since cell numbers assessed by measurement of cytosolic LDH activity as well as cell proliferation assessed by BrdU incorporation remained unchanged during the course of the experiments (data not shown). IL-1β-induced ERK1/2 activation in cells plated onto TcP or onto ECM-coated plates was completely abrogated in the presence of the IL-1 receptor antagonist, IL-1RA, demonstrating that all responses were mediated by IL-1R1 (Fig. 3B). All responses were abrogated by heat-treating (90 °C for 30 min) the IL-1β preparation, demonstrating the purity of the recombinant IL-1β preparation used.

### Integrin β1 mediates ECM-dependent actions of IL-1β

**Fig. 4:**
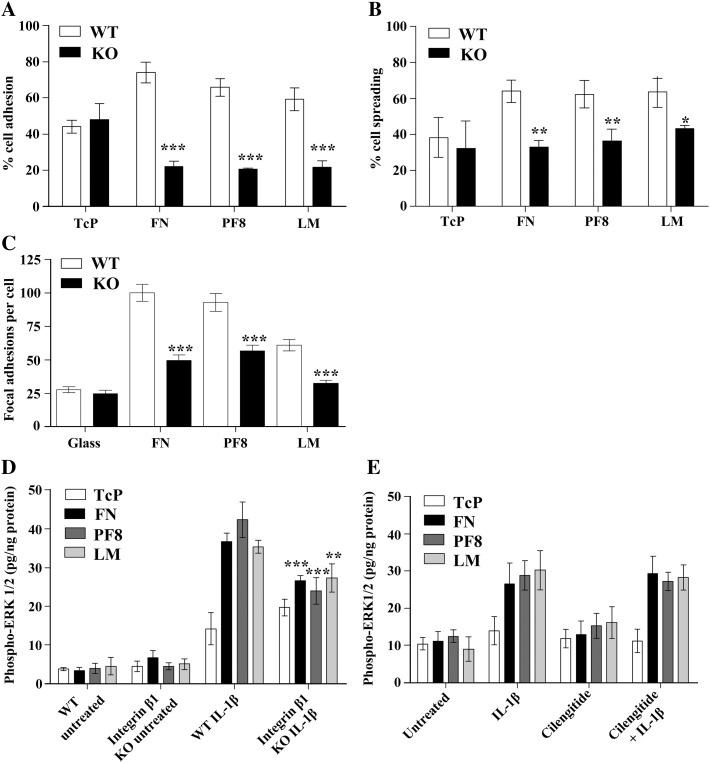
Integrin β1 mediated glial attachment and spreading on ECM, and ECM effects on IL-1β-induced ERK1/2 activation. Integrin β1 knockout (KO) glial cells showed significantly less adhesion (A) and spreading (B) than wild-type (WT) cells using a two-way ANOVA with Bonferroni *post-hoc* tests to compare all data where *P* < 0.001 ***, *P* < 0.01 ** and *P* < 0.05 *. C. Integrin β1 KO cells had significantly fewer focal adhesions on FN, PF8 and LM than WT cells, according to a one-way ANOVA with Tukey *post-hoc* tests where *P* < 0.001 ***. D. Integrin β1 KO cells had significantly less ERK1/2 activation, following IL-1β treatment (10 ng/mL, 30 min), compared to WT cells on FN, PF8 and LM, where *P* < 0.001 *** and *P* < 0.05 **, according to a two-way ANOVA with Bonferroni *post-hoc* tests to compare all values. E. However, pre-treatment with the αv integrin inhibitor, cilengitide (10 μM, 30 min), did not significantly affect ERK1/2 activation following IL-1β treatment (10 ng/mL, 30 min) compared to cells treated with IL-1β alone. Levels of phosphorylated ERK1/2 in D and E have been corrected by protein assay (expressed as pg/ng proteins) to correct for differences in cell numbers between WT and KO cultures. Data presented are the mean ± SD of three independent experiments.

Integrin β1 knockout (KO) cells adhered significantly less to FN, PF8 and LM than WT cells (Fig. 4A), in agreement with effects of the integrin β1 blocking antibody on cell adhesion (Fig. 2B). Adhesion to TcP was unchanged in integrin β1 KO cells compared to WT cells, demonstrating that integrin β1 was not involved in non-specific cell adhesion to an inert substrate. Integrin β1 also mediated cell spreading, since integrin β1 KO cells showed significantly less spreading (Fig. 4B) and formed significantly fewer focal adhesions (Fig. 4C) on FN, PF8 and LM compared to WT cells. Importantly, IL-1β-induced ERK1/2 activation was significantly lower in integrin β1 KO cells than in WT cells (Fig. 4D), implying that the effect of ECM on IL-1β-induced ERK1/2 activation is mediated by cell adhesion via integrin β1. However, the effect of ECM on IL-1β-induced ERK1/2 activation was not affected by treatment of cells with cilengitide (Fig. 4E), demonstrating that integrin αv was not critically involved in this response.

### IL-1β-induced RhoA/ROCK deactivation and loss of focal adhesions are ECM-dependent

**Fig. 5:**
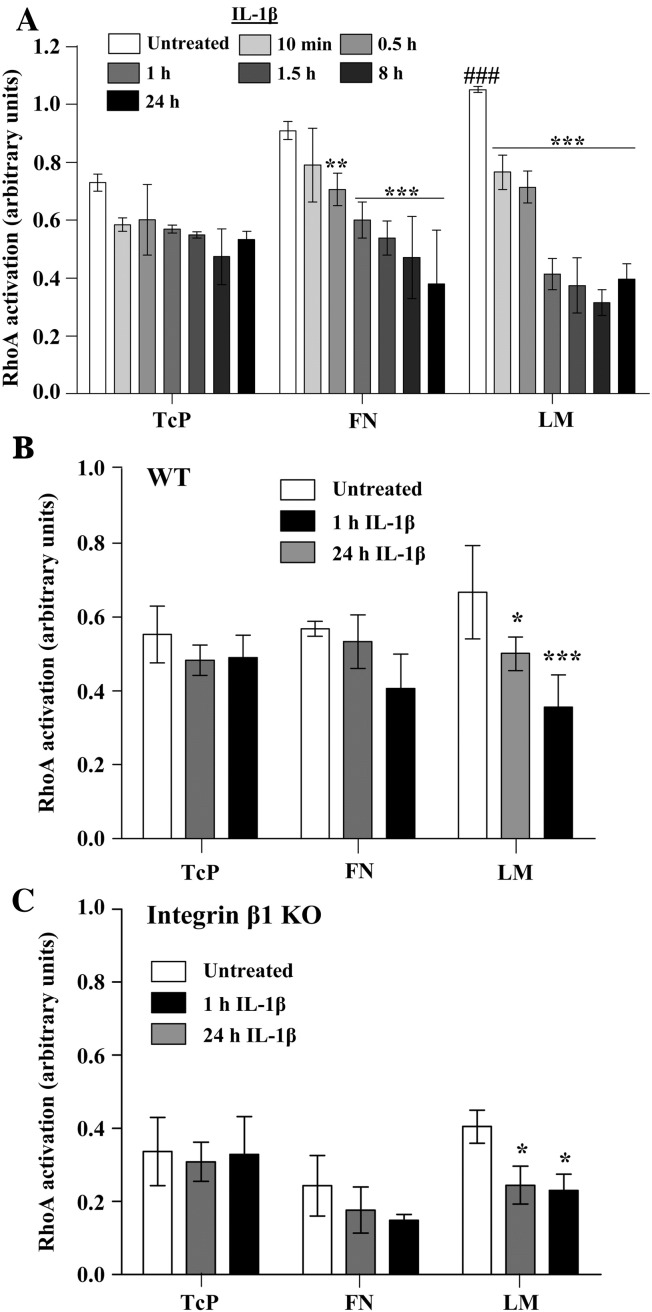
IL-1β-induced RhoA deactivation was ECM dependent. A. Rat astrocytes were treated with IL-1β (10 ng/mL from 10 min to 24 h) and RhoA activation was assessed by ELISA. IL-1β significantly inhibited RhoA activation compared to untreated cells on FN and LM, but not on TcP, where *P* < 0.01 ** and *P* < 0.001 ***. Cells attached to LM had significantly higher RhoA activation than cells on TcP, where *P* < 0.001 ###. B. Similarly, in WT murine cells, activation of RhoA was significantly inhibited by IL-1β treatment (10 ng/mL for 1 or 24 h) compared to untreated cells attached to LM, where *P* < 0.05 * and *P* < 0.001 ***. C. Integrin β1 KO murine cells had lower levels of RhoA activation compared to WT cells, but IL-1β treatment (10 ng/mL for 1 h or 24 h) significantly reduced RhoA activation further on LM compared to untreated cells, where *P* < 0.05 *. All data were analysed by two-way ANOVA with Bonferroni *post-hoc* tests. Data presented are the mean ± SD of three independent experiments.

IL-1β has been reported to deactivate RhoA in astrocytes (John et al., 2004), and RhoA is known to be activated by attachment to ECM (Wang et al., 2005). Therefore we investigated whether the ECM regulates IL-1β-induced RhoA deactivation and subsequent loss of focal adhesions. RhoA activation was significantly increased in cells seeded onto LM compared to TcP (Fig. 5A), and activation was higher in cells attached to FN compared to TcP, although this was not significant. Importantly, IL-1β induced RhoA deactivation in a time-dependent manner when cells were attached to ECM molecules, but had no effect on the levels of RhoA activity in cells seeded onto TcP, demonstrating that the ECM is required for IL-1β-induced RhoA deactivation. This effect was completely abrogated in the presence of IL-1RA (data not shown). Using murine astrocytes, we found that the levels of active RhoA were strongly reduced in integrin β1 KO cells compared to WT cells, but IL-1β treatment still reduced RhoA activation in integrin β1 KO cells compared to untreated integrin β1 KO cells (Fig. 5B and 5C).

**Fig. 6:**
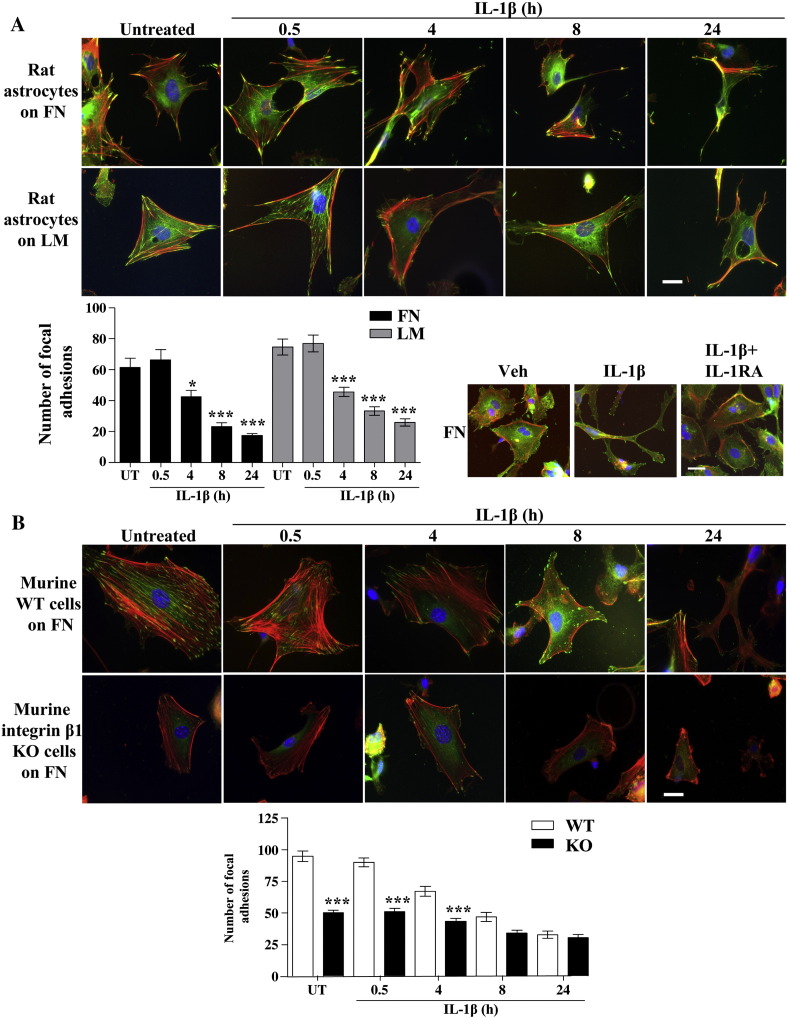
IL-1β treatment induced loss of focal adhesions when cells were attached to ECM. A. Rat astrocytes were treated with IL-1β (10 ng/mL), in the absence or the presence of IL-1RA (10 μg/mL), over a time course of 24 h, and stained with an antibody against vinculin (green), phalloidin to detect actin stress fibers (red) and DAPI nuclear staining (blue). Focal adhesions were quantified using Image J software, and IL-1β treated cells showed a significant reduction in the number of focal adhesions compared to untreated cells when attached to FN or LM, according to one-way ANOVAs with Tukey *post-hoc* tests to compare all data where *P* < 0.05 * and *P* < 0.001 ***. B. Integrin β1 KO cells showed significantly fewer focal adhesions than WT cells when untreated or treated with IL-1β (10 ng/mL, for 0.5 h or 4 h), using a two-way ANOVA with Bonferroni *post-hoc* tests to compare all data sets, where *P* < 0.001 ***. Scale bars represent 20 μm and images are representative of three independent experiments. Data presented are the mean ± SD of three independent experiments.

**Fig. 7:**
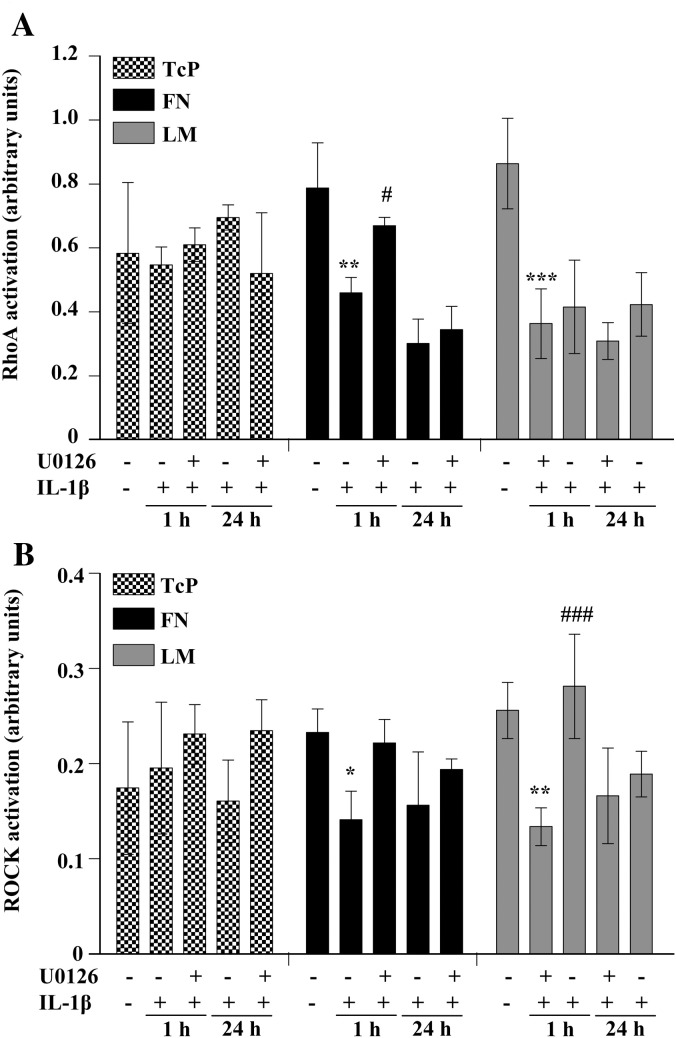
MEK inhibition partially prevented IL-1β-induced RhoA/ROCK inhibition. A. Astrocytes were treated with IL-1β (10 ng/mL, 1 h) and RhoA activation was assessed by ELISA. IL-1β treatment significantly inhibited RhoA activation compared to untreated cells attached to FN and LM, where *P* < 0.01 ** and *P* < 0.001 ***. Astrocytes on FN had significantly higher RhoA activation when treated with the MEK inhibitor, U0126 (20 μM, 30 min), prior to IL-1β treatment compared to cells treated with IL-1β alone, where *P* < 0.05 #. B. ROCK activation was also assessed by ELISA. IL-1β treatment (10 ng/mL, 1 h) significantly inhibited ROCK activation when cells were attached to FN or LM, where *P* < 0.05 * and *P* < 0.01 **. ROCK activation was significantly higher when cells were pre-treated with U0126 (20 μM, 30 min) before IL-1β treatment, on LM where *P* < 0.001 ### compared to cells treated with IL-1β alone. Data were analysed by two-way ANOVA with Bonferroni *post-hoc* tests to compare all data. Data presented are the mean ± SD of three independent experiments.

Since RhoA is involved in formation of focal adhesions and regulation of the cytoskeleton (Hall, 2005), we investigated whether IL-1β-induced RhoA deactivation affected cell morphology. Indeed, astrocytes seeded on FN and LM showed a significant loss of focal adhesions following IL-1β treatment from 4 to 24 h compared to untreated cells (Fig. 6A), coincident with RhoA inhibition (Fig. 5A). Loss of focal adhesions was accompanied by a dramatic change in morphology whereby cells changed from a large, flat shape with a well organised cytoskeleton, to a retracted, stellate morphology with a disordered cytoskeleton. This morphology was very similar to that obtained using ROCK inhibitors, H1152 or Y27632 (data not shown), and are in agreement with our data showing that IL-1β deactivates RhoA and ROCK (Figs. 5 and 7B, respectively). IL-1β-induced change of astrocytic morphology on ECM was completely reversed in the presence of IL-1RA (Fig. 6A). Cells plated onto BSA-coated glass coverslips displayed non-functional focal adhesions that were not altered by IL-1β treatments (data not shown). Furthermore, integrin β1 KO cells formed fewer focal adhesions than WT cells, but treatment with IL-1β still led to significant loss of focal adhesions and morphological changes, similar to those observed in WT cells (Fig. 6B).

### IL-1β-induced RhoA/ROCK inhibition and morphological changes are dependent on the MEK/ERK1/2 pathway

MEK (upstream kinase of ERK1/2) has been reported to induce similar morphological changes to those seen in our experiments (Pawlak and Helfman, 2002). We therefore investigated whether inhibiting the MEK/ERK1/2 pathway reversed the effects of IL-1β on RhoA activity and cell morphology. Pre-treatment of cells plated on FN with the MEK inhibitor U0126 (specific inhibition was confirmed by Western blot analysis, data not shown), significantly prevented IL-1β-induced RhoA inhibition compared to cells without U0126 pre-treatment (Fig. 7A). A similar effect was observed in cells plated on LM, although this was not significant. To further confirm that the MEK/ERK1/2 pathway regulates RhoA activity, we investigated the effect of U0126 on IL-1β-induced ROCK deactivation. We found that IL-1β significantly inhibited ROCK activation in cells plated onto FN or LM, but had no effect in cells seeded onto TcP (Fig. 7B). U0126 pre-treatment prevented IL-1β-induced ROCK inhibition in cells plated onto LM, with a similar effect in cells on FN, but had no effect on ROCK activity in cells plated onto TcP. These data demonstrate that IL-1β-induced RhoA/ROCK deactivation was MEK-dependent and occurred only in cells plated onto ECM molecules.

**Fig. 8:**
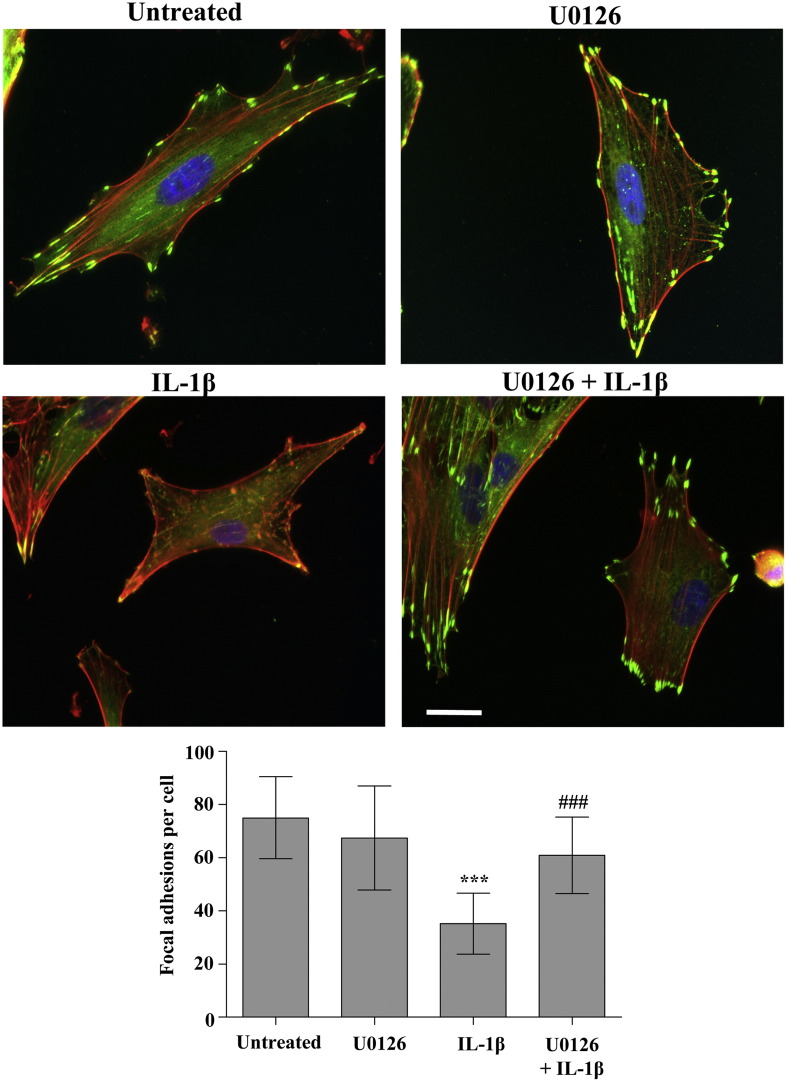
MEK inhibition prevented IL-1β-induced loss of focal adhesions. Astrocytes were pre-treated with U0126 (20 μM, 30 min), and/or treated with IL-1β (10 ng/mL, 24 h), then stained with an antibody against vinculin (green), phalloidin to detect actin stress fibers (red) and DAPI nuclear staining (blue). Astrocytes treated with IL-1β had significantly fewer focal adhesions than untreated cells seeded onto FN where *P* < 0.001 ***. Yet cells pre-treated with U0126 and then treated with IL-1β had significantly more focal adhesions than cells treated with IL-1β alone where *P* < 0.001 ###, using a one-way ANOVA with Tukey *post-hoc* tests to compare all data sets. Data presented are the mean ± SD of three independent experiments. Scale bars represent 20 μm and images are representative of three independent experiments.

We then investigated whether U0126 could alter astrocyte morphology induced by IL-1β treatment. IL-1β-induced loss of focal adhesions was significantly prevented by pre-treatment with U0126, whilst the inhibitor alone had no effect on the number of focal adhesions (Fig. 8). Finally, immunocytochemistry clearly showed that astrocytes treated with U0126 prior to IL-1β retained a spread morphology with a well organised cytoskeleton compared to degradation of the cytoskeleton and stellation seen in the absence of U0126.

## Discussion

The ECM of the CNS is essential for maintenance of brain homeostasis, but undergoes structural changes following acute injury. This causes exposure of brain cells to vascular ECM that undergo degradation by proteases (Fukuda et al., 2004), as well as *de novo* ECM molecules expressed and deposited in the brain blood vessels and parenchyma (Tate et al., 2007). To date, no studies have investigated the role of cell attachment to the ECM on IL-1 actions in astrocytes. We report here a novel regulatory mechanism by which cell adhesion to the ECM is essential for IL-1β-induced signalling pathways in astrocytes, leading to the development of a reactive phenotype *in vitro*. Firstly, we showed strong adhesion of astrocytes to FN, PF8 and LM (Fig. 1A), and demonstrated that integrins were actively engaged in cellular adhesion to ECM molecules, in that integrin β1 mediated adhesion to FN, PF8 and LM, integrin α4 mediated adhesion to FN, and integrin α5 and αv mediated adhesion to FN and PF8 (Fig. 2). Our data show that seeding astrocytes onto ECM molecules promotes cell spreading (Fig. 1B) and formation of focal adhesions (Fig. 1C), accompanied by cell enlargement and a well organised cytoskeleton.

Consequently, we demonstrated that IL-1β-induced ERK1/2 activation was strongly enhanced and sustained in astrocytes adhered to ECM molecules (Fig. 3A). These effects were dependent on integrin β1, as demonstrated using inducible integrin β1 KO cells (Fig. 4D), but were independent on integrin αv (Fig. 4E). Although ERK1/2 activation downstream of integrin signalling has been reported previously, no integrin-dependent ERK1/2 activity was measured in our study since increased IL-1β-induced ERK1/2 activation in cells on ECM was completely abrogated in the presence of IL-1RA. These data clearly demonstrate that cellular adhesion to ECM modifies some signalling mechanisms leading to enhancement of IL-1-dependent ERK1/2 activity. Furthermore, we showed that IL-1β induced deactivation of the RhoA/ROCK signalling pathway in an ECM-dependent manner (Fig. 5). A previous study demonstrated that IL-1β-induced deactivation of the RhoA/ROCK signalling pathway mediates astrocytic morphological changes from a flat elongated to a stellar morphology, and was reported as reactive astrogliosis (John et al., 2004). In our study, we demonstrated that the deactivation of the RhoA/ROCK pathway by IL-1β, concomitant with a dramatic loss of focal adhesions and change to stellar cellular morphology, was dependent on the ECM (Fig. 6A). The relevance of IL-1-induced reactive astrocyte phenotype *in vitro* to astrogliosis *in vivo* remains to be fully addressed. However similar astrocytic morphological features (cellular flattening, elongation and stellation) were observed *in vivo* (Giulian et al., 1986; Herx and Yong, 2001) and *in vitro* (John et al., 2004), suggesting that these mechanisms are common. Our data confirm that IL-1β-induced RhoA/ROCK deactivation is a key signalling event in the development of reactive astrocyte phenotype, and suggest that the ECM may be an important regulator of IL-1β-induced astrogliosis. The effect of IL-1β-induced astrogliosis was absent in integrin β1 KO cultures. To date, no studies have demonstrated a role for integrin β1 in the regulation of astrogliosis after brain injury, but our data suggest that cell adhesion to the surrounding ECM network via integrin β1 is a key step for the induction of astrogliosis and possibly the migration of reactive astrocytes to the site of injury to form the glial scar.

We further demonstrated that the inhibition of the RhoA/ROCK pathway and the induction of reactive phenotype were dependent on MEK activity, since pharmacological inhibition of MEK strongly reduced the effect of IL-1β on RhoA/ROCK deactivation (Fig. 7). MEK inhibition could not rescue levels of RhoA/ROCK activation after a 24 h IL-1β treatment, implying that there may be a critical early wave of IL-1β-induced MEK activation that deactivates RhoA/ROCK at the early time-point. However MEK inhibition did prevent loss of focal adhesions induced by 24 h IL-1β treatment (Fig. 8), implying that a level of cross-talk between the MEK and RhoA/ROCK signal pathways still occurs at this later time-point. The precise mechanism by which the MEK/ERK1/2 and RhoA/ROCK signalling pathways interact remains unclear (Zuckerbraun et al., 2003; Laboureau et al., 2004), and although our data suggest that ERK1/2 regulates RhoA, we also found that inhibition of RhoA using H1152 induces ERK1/2 activation (data not shown). The lack of effect of MEK inhibition on IL-1β-induced RhoA/ROCK deactivation at 24 h also suggest that the MEK/ERK1/2 pathway may become redundant, and that alternative signalling mechanisms may take place at this time.

Our experiments have focused on the role of RhoA/ROCK in astrocyte morphology and focal adhesion formation, but other signalling pathways might also be involved. Src kinase has been shown to regulate cell morphology in a MEK-dependent manner (Pawlak and Helfman, 2002), and Src kinase can be activated by S100β, which is a marker of astrogliosis (Brozzi et al., 2009). RhoA is a multifunctional molecule that has been reported to control many other signalling elements such as c-Jun N-terminal kinase, p38 and nuclear factor kappa B [see (Hall, 2005) for review]. This may explain why MEK inhibition significantly prevented loss of focal adhesions following 24 h IL-1β treatment, but did not significantly alter RhoA or ROCK levels at this time-point.

In conclusion, our data demonstrate that the ECM is required for the activation of MEK/ERK1/2 and RhoA/ROCK signalling pathways triggered by IL-1β in astrocytes, and that attachment to ECM molecules, which become available to astrocytes following acute CNS injury, may be a critical event in the process of reactive astrocytic phenotype triggered by IL-1.

## Experimental methods

### Extracellular matrix proteins and plate coating

Recombinant FBN1 protein fragment 8 (PF8) was produced in-house as previously reported (Cain et al., 2005). Human plasma fibronectin (FN) was purchased from Chemicon, and natural mouse laminin 111 (LM) was purchased from Invitrogen.

Tissue culture plates (Corning) were pre-coated with ECM molecules at 100 nM in 9.5 mM phosphate buffered saline containing Ca^2+^ and Mg^2+^ cations (PBS^+^) (Invitrogen) for 1 h at room temperature or at 4 °C overnight. Wells were blocked with 0.22 μM filtered 0.1% bovine serum albumin (BSA) (Invitrogen) in PBS without Ca^2+^ and Mg^2+^ cations (PBS^−^) at room temperature for 1 h, and then washed in PBS^−^ three times before seeding cells.

### Cultures of rat astrocytes and inducible integrin β1 knockout (KO) mouse astrocytes

Mixed glial cell cultures were prepared from 0- to 2-day-old Sprague Dawley rat pups as described previously (Summers et al., 2009) and grown in a humidified incubator at 37 °C with 5% CO_2_ until confluency (14–20 days *in vitro* (DIV)). Cultures were then shaken at 300 rpm for 2 h and the culture medium containing microglia was discarded. The astrocytic monolayer was washed with PBS^−^ and then trypsinised with 1X trypsin (Invitrogen) for 3–5 min at 37 °C. Trypsin was quenched with DMEM (BioWhittaker) containing 1% penicillin/streptomycin and 10% FCS and cells were collected by centrifugation at 2000 rpm for 5 min. The pellet was resuspended in DMEM, and cells were seeded onto ECM-coated or uncoated tissue culture plates (cell density adjusted according to experiments) and incubated at 37 °C overnight. The composition of the mixed glial cultures was confirmed by immunofluorescence to be 97% astrocytes and 3% microglia.

Conditional integrin β1 knockout (KO) mice were a gift from Prof. Streuli (University of Manchester). These mice were obtained by crossing Cre recombinase-expressing mice with mice carrying *integrin* *β1* alleles flanked by loxP sites. Individual cultures were prepared from the whole brain of separate embryos at 16–18 days of embryonic development, and the corresponding skull was used for Cre genotyping analysis. Each skull was dissolved into lysis buffer (containing 100 mM Tris–HCl pH 8.5, 5 mM EDTA pH 8.0, 0.2% SDS, 200 mM NaCl and 100 μg/mL proteinase K in H_2_O) in a rotary device at 55 °C for 48 h. Samples were diluted 1:5 with DNase free water, and PCR was performed with 50 mM MgCl_2_, 10× PCR buffer, 25 μM dNTPs and 10 μM forward and reverse primers (Fwd, 5′-AACCTGGATAGTGAAACAGGGGC-3′; Rev, 5′-GGAACCGACTTGACGTAGCCAGC-3′). Confluent Cre-positive mixed glial cultures were treated with 100 nM tamoxifen (Sigma) for 72 h prior to trypsinisation and plating onto ECM-coated tissue culture plates. Successful deletion of integrin β1 was confirmed by Western blot analysis (data not shown), and tamoxifen-treated Cre-negative cultures were used as corresponding wild-type (WT) cultures.

### Culture treatments

Cultures were treated with 10 ng/mL rat recombinant IL-1β (National Institute for Biological Standards and Control, NIBSC, UK) from 5 min to 24 h, in the absence or presence of 10 μg/mL IL-1RA (NIBSC). RGD peptides and control RAD peptides (Bachem) were used at 100 μM for 30 min prior to cell seeding. Integrin-blocking antibodies were used at 10 μg/mL including: anti-mouse/rat CD29 (integrin β1) monoclonal antibody (BioLegend), anti-mouse/rat CD49e (integrin α5) monoclonal antibody (BioLegend), and mouse anti-human CD49d (integrin α4) monoclonal antibody (AbD Serotec), applied for 30 min prior to cell seeding. A cyclic RGD peptide inhibitor of αv integrin (cilengitide) was kindly provided by S. Goodman (Merck KGaA, Germany) and used at 10 μM concentration. Cells were treated with 20 μM of the MEK inhibitor U0126 (Promega) dissolved in DMSO, 30 min prior to IL-1β treatment.

### Cell adhesion and spreading assays

Cell adhesion and cell spreading assays were performed using crystal violet staining and assessment of cellular morphology under light microscopy respectively, as previously reported (Summers et al., 2009).

### Immunofluorescence

Cells were seeded onto ECM-coated glass coverslips at a density of 1.0 × 10^5^ cells/mL and incubated overnight at 37 °C. Cells were then fixed for 10 min with 4% paraformaldehyde (BDH Laboratories) and 4% sucrose (BDH Laboratories) in PBS. Cells were permeabilised with 0.5% (w/v) triton X-100 in PBS^−^ for 5 min and quenched with NH_4_Cl_2_ for 10 min. To detect focal adhesions, cells were incubated with a mouse monoclonal hVIN-1 anti-vinculin antibody (Abcam, 1:400 dilution in PBS^−^) at room temperature for 1 h followed by incubation with a rabbit anti-mouse fluorescein-conjugated secondary antibody (DAKO, 1:40 dilution in PBS^−^), and Texas Red®-conjugated phalloidin (Invitrogen, 1:400 dilution in PBS^−^) to detect actin stress fibers, for 1 h at room temperature. Coverslips were mounted with ProLong Gold antifade reagent with DAPI (Invitrogen), and washed three times in PBS^−^ before addition of each reagent. Images were acquired on a Delta Vision RT (Applied Precision) restoration microscope using a [60x 1.42 Plan Apo] objective. The images were collected using a Coolsnap HQ (Photometrics) camera with a Z optical spacing of 0.2 μm, and analysed using Image J 1.37v (National Institutes of Health).

### Measurement of ERK1/2 activation

Cells seeded onto ECM-coated 12-well plates (5.0 × 10^5^ cells/well) and treated with IL-1β (10 ng/mL) for 5, 15, 30, 60 or 120 min were then lysed in lysis buffer (1 mM EDTA, 0.5% Triton X-100, 5 mM NaF, 6 M urea, protease inhibitor cocktail, 100 μM PMSF, 2.5 mM disodium pyrophosphate and 1 mM sodium orthovanadate in PBS, pH 7.2–7.4). DuoSet® IC Human/mouse/rat phospho-ERK1/2 ELISA was used according to the manufacturer's instructions (R&D Systems), with a detection limit of 625 pg/mL. Levels of phosphorylated ERK1/2 in the cell lysates were expressed as pg/mL.

### Quantification of RhoA//ROCK activation

Cells seeded onto ECM-coated 6-well plates (5.0 × 10^5^ cells/well) and treated with IL-1β (10 ng/mL) for 0.5, 1, 1.5, 8 or 24 h. were then lysed and assayed for RhoA activation using a G-LISA™ RhoA Activation Assay Biochem Kit™ (Cytoskeleton/Universal Biologicals), according to the manufacturer's instructions. ROCK activity was assayed using a 96-well ROCK activity assay kit (Cell Biolabs) according to the manufacturer's instructions.

### Quantification of focal adhesions

Focal adhesions were quantified using Image J software. The raw images of vinculin staining were adjusted for brightness to remove all background staining in order to visualise focal adhesions only. The dotted immunostaining corresponding to focal adhesions was automatically counted to produce a quantitative value per cell. Brightness was adjusted equally for all images.

### Statistical analysis

Data were analysed using GraphPad Prism version 4.0 for Microsoft Windows. Cell spreading assays and immunofluorescent data were analysed by a one-way ANOVA with Tukey *post-hoc* test. Cell adhesion data and RhoA ELISA data from the IL-1 timecourse were analysed by two-way ANOVA with Bonferroni *post-hoc* test to compare untreated cells to treated cells. Data from phospho-ERK1/2 ELISA, integrin β1 KO cells and RhoA/ROCK ELISA kits with U0126 were compared by two-way ANOVA with Bonferroni *post-hoc* test. The level of significance was represented as *P < 0.05, **P < 0.01 and ***P < 0.001.
